# Crystal structure of 6-(4-chloro­phen­yl)-6a-nitro-6a,6b,8,9,10,12a-hexa­hydro-6*H*,7*H*-spiro[chromeno[3,4-*a*]indolizine-12,11′-indeno­[1,2-*b*]quinoxaline]

**DOI:** 10.1107/S2056989019000975

**Published:** 2019-01-25

**Authors:** S. Syed Abuthahir, M. NizamMohideen, V. Viswanathan, D. Velmurugan, J. Nagasivarao

**Affiliations:** a PG & Research Department of Physics, The New College (Autonomous), Chennai 600 014, Tamil Nadu, India; b Department of Biophysics, All India Institute of Medical Science, New Delhi 110 029, India; c CAS in Crystallography and Biophysics, University of Madras, Chennai 600 025, India; d GVK Biosciences Pvt. Ltd, Hyderabad 500 076, India

**Keywords:** crystal structure, spiro compounds, cyclo­addition, piperidine, pyran, pyrrolidine, cyclo­pentene, hydrogen bonding, C—H⋯π inter­actions

## Abstract

The title compound, C_35_H_27_ClN_4_O_3_, crystallized with two independent mol­ecules (*A* and *B*) in the asymmetric unit. In both mol­ecules, the pyran and pyridine rings adopt envelope and chair conformations, respectively. The conformation of the pyrrolidine and cyclo­pentene rings differ in the two mol­ecules; twisted and flat, respectively, in mol­ecule *A*, but envelope and twisted, respectively, in mol­ecule *B*. In both mol­ecules, there is a C—H⋯N intra­molecular hydrogen bond present. In both mol­ecules, the oxygen atoms of the nitro groups are disordered as is the chlorine atom in mol­ecule *B*. In the crystal, the *B* mol­ecules are linked by C—H⋯O hydrogen bonds, forming *–B–B–B–* chains along [010], and by C—H⋯π inter­actions. The *A* and *B* mol­ecules are also linked by a number of C—H⋯π inter­actions, resulting in the formation a supra­molecular three-dimensional structure. In mol­ecule *A*, the nitro group oxygen atoms are disordered over two positions with refined occupancy ratios of the nitro group oxygen atoms O3*A* and O4*A* in 0.59 (2):0.41 (2) while in molecule *B* one of the nitro O atoms is disordered over two positions with a refined occupancy ratio of 0.686 (13):0.314 (13) and the chlorine atoms is disordered over two positions with a refined occupancy ratio of 0.72 (3):0.28 (3).

## Abstract 2

The title compound crystallized with two independent mol­ecules (*A* and *B*) in the asymmetric unit. They differ essentially in the conformation of the pyrrolidine and cyclo­pentene rings; respectively, twisted and flat in mol­ecule *A*, but envelope and twisted in mol­ecule *B*.

## Chemical context   

Nitro­gen-containing heterocyclic compounds are reported to possess a diverse range of biological activities such as anti­microbial, anti­tumor and anti-inflammatory (Syed Abuthahir *et al.*, 2019 ▸; Thirunavukkarsu *et al.*, 2017 ▸) properties. Spiro compounds are often encountered in many pharmacologically active alkaloids (NizamMohideen *et al.*, 2009*c* ▸; Cravotto *et al.*, 2001 ▸). The cornerstone for cyclo­addition reactions, nitro­nes are excellent spin-trapping and highly versatile synthetic inter­mediates (Bernotas *et al.*, 1996 ▸; NizamMohideen *et al.*, 2009*b* ▸). Highly substituted spiro compounds result from the 1,3-dipolar cyclo­addition of exocylic olefins with nitro­nes; these spiro compounds have also been transformed into complex heterocycles (Hossain *et al.*, 1993 ▸; NizamMohideen *et al.*, 2009*a* ▸). Recognizing the importance of such compounds in drug discovery and our ongoing research into the construction of novel heterocycles has prompted us to investigate the title compound: we report herein the synthesis and the crystal structure. ▸).
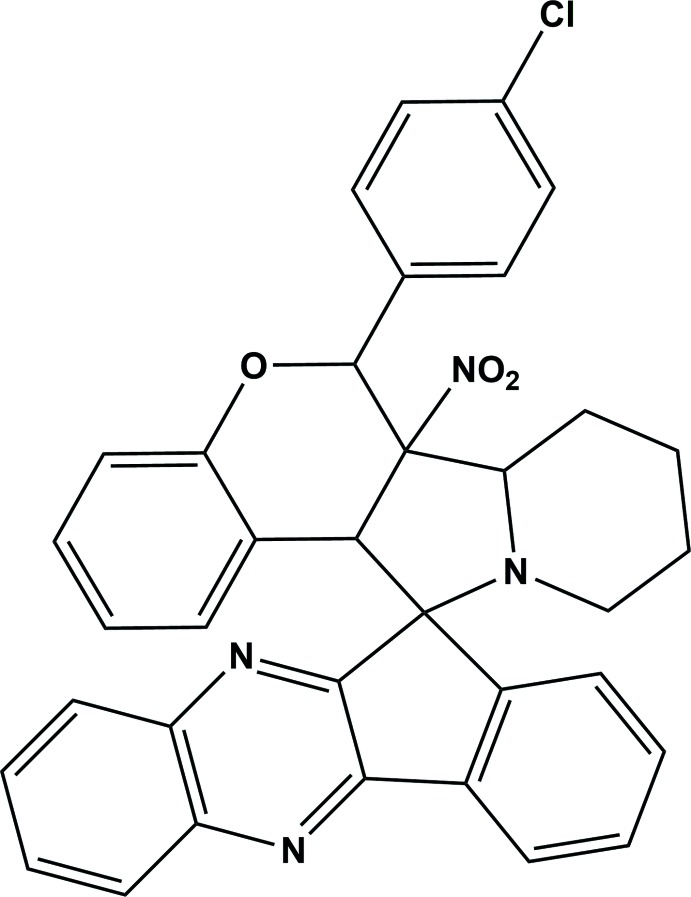


## Structural commentary   

**Figure 1:**
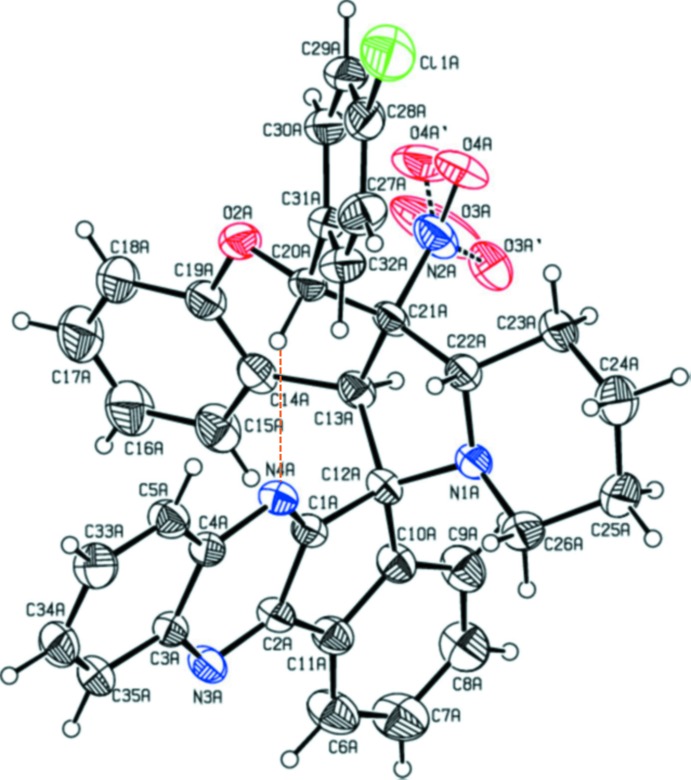
View of the mol­ecular structure of mol­ecule *A* of the title compound, with the atom labelling. Displacement ellipsoids are drawn at the 30% probability level. The intra­molecular C—H⋯N contact (Table 1 ▸) is shown as an orange dashed line.

The mol­ecular structures and conformation of the two independent mol­ecules, *A* and *B*, in the asymmetric unit are shown in Figs. 1 ▸ and 2 ▸, respectively. In both mol­ecules, the oxygen atoms of the nitro groups are disordered as is the chlorine atom in mol­ecule *B*. For all further discussion only the major components of the disordered atoms will be considered. The bond lengths and angles are close to those reported for similar compounds (Devi *et al.*, 2013*a* ▸,*b* ▸; Syed Abuthahir *et al.*, 2019 ▸). In both mol­ecules there is an C—H⋯N intra­molecular hydrogen bond present enclosing an *S*(7) ring motif (Fig. 1 ▸ and Table 1

**Figure 3:**
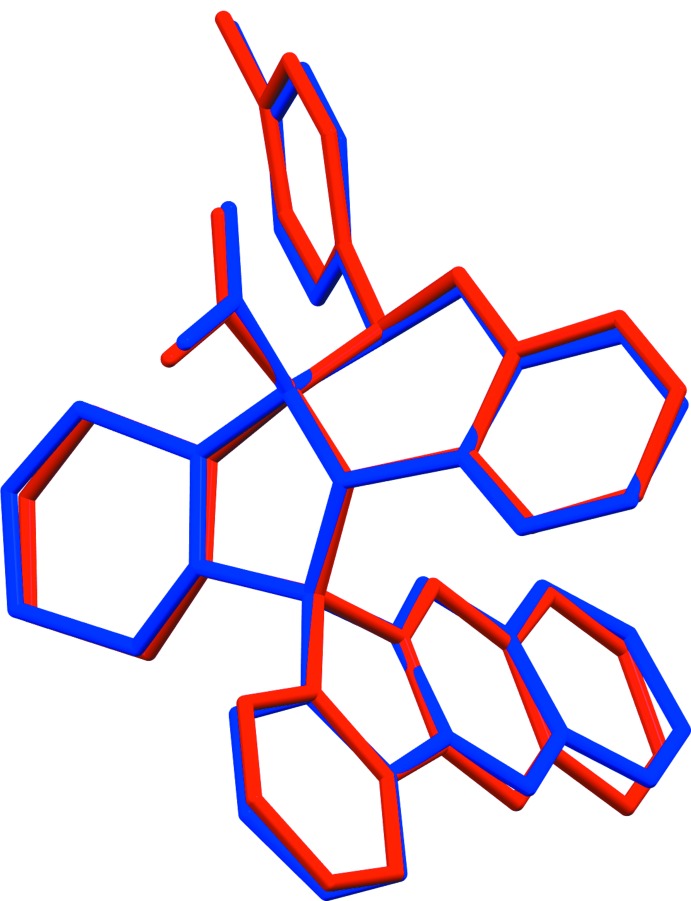
A view of the mol­ecule overlay of inverted mol­ecule *B* (red) on mol­ecule *A* (blue), with an r.m.s. deviation of 0.208 Å. The H atoms have been omitted for clarity.

The overall conformation of the two mol­ecules is very similar, as seen from the mol­ecule overlay figure (Fig. 3 ▸), calculated and drawn using *Mercury* (Macrae *et al.*, 2008 ▸). The essential difference concerns the conformations of the pyrrolidine (N1/C12/C13/C21/C22) and cyclo­pentene (C1/C2/C10–C12) rings. In mol­ecule *A* the former ring has an envelope conformation with atom C12*A* as the flap, while in mol­ecule *B* this ring has a twisted conformation on the N1*B*—C12*B* bond. The cyclo­pentene ring is twisted on the C12*A*—C1*A* bond in mol­ecule *A* but is flat in mol­ecule *B*. The pyran rings (O2/C19/C14/C13/C21/C20) in both mol­ecules have envelope conformations with atom C20 as the flap, and the piperidine rings in both mol­ecules (N1/C22–C26) have chair conformations.

Chlorine atoms Cl1*A* and Cl2 deviate from the mean plane of the benzene rings to which they are attached (respectively C27*A*–C32*A* and C27*B*–C32*B*) by 0.006 (1) and 0.053 (16) Å, respectively. The mean plane of the five-membered pyrrolidine ring (N1/C12/C13/C21/C22) is inclined to the mean plane of the cyclo­pentene ring (C1/C2/C10–C12) by 87.30 (13) and 88.41 (11)° in mol­ecules *A* and *B*, respectively. The benzene rings C27–C32 and C14–C19 are inclined to each other by 58.13 (13)° in mol­ecule *A* and 57.13 (11)° in mol­ecule *B*, while benzene rings C6–C11 and C3–C5/C33–C35 are inclined to each other by 10.20 (13)° in mol­ecule *A* and 4.08 (13)° in mol­ecule *B*. The mean plane of the pyrrolidine ring (N1/C12/C13/C21/C22) makes a dihedral angle with the mean plane of the pyran ring (O2/C13/C14/C19–C21) of 34.6 (2)° in mol­ecule *A* and 29.65 (10)° in mol­ecule *B*, and is inclined to the piperidine ring mean plane (N1/C22–C26) by 15.69 (12)° in mol­ecule *A* and 12.36 (11)° in mol­ecule *B*. The mean planes of the pyran and piperidine rings are inclined to each other by 37.06 (11) and 29.49 (10)° in mol­ecules *A* and *B*, respectively. The mean plane of the pyrazine ring (N3/N4/C1/C2/C3/C4) makes a dihedral angle with the mean plane of the pyran ring (O2/C13/C14/C19–C21) of 63.42 (19)° in mol­ecule *A* and 72.64 (10)° in mol­ecule *B*. It is inclined to the pyrrolidine ring mean plane (N1/C12/C13/C21/C22) by 88.11 (1)° in mol­ecule *A* and 86.69 (11)° in mol­ecule *B* and is inclined to the piperidine ring mean plane (N1/C22–C26) by 77.24 (11)° in mol­ecule *A* and 82.97 (11)° in mol­ecule *B*.

## Supra­molecular features   

**Figure 4:**
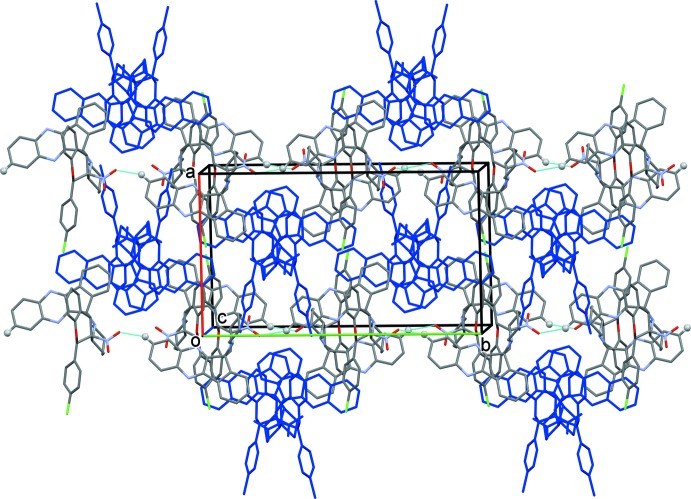
The crystal packing of the title compound viewed along the *c* axis. The inter­linking of *B* mol­ecules *via* C—H⋯O hydrogen bonds (see Table 1 ▸ for details) generates chains running along the [010] direction. The *A* mol­ecules are shown in blue and the hydrogen bonds are shown as dashed lines. For clarity, H atoms not involved in the various inter­molecular inter­actions have been omitted.

In the crystal, the *B* mol­ecules are linked by C—H⋯O hydrogen bonds, forming chains propagating along the *b*-axis direction (Table 1 ▸ and Fig. 4 ▸). The *B* mol­ecules are also linked by C—H⋯π inter­actions, and are linked to the *A* mol­ecules by C—H⋯π inter­actions (Table 1 ▸). The result of these inter­molecular inter­actions is the formation of a supra­molecular three-dimensional structure (Table 1 ▸ and Fig. 4 ▸).

## Database survey   

A search of the Cambridge Structural Database (CSD, Version 5.39, August 2018; Groom *et al.*, 2016 ▸) for the 6′-(4- phen­yl)-6a’-hexa­hydro-2*H*,6′*H*,6b’ *H*-spiro­[benzo­pyrano[3,4-*a*]indolizin]-2-one skeleton yielded hits for two mol­ecules similar to the title compound: namely 6-(4-meth­oxy­phen­yl)-6a-nitro-6,6a,6 b,7,8,9,10,12a-octa­hydro­spiro­[chromeno[3,4-*a*]indolizine-12,3-indolin]-2-one (CSD refcode AFONEQ; Devi *et al.*, 2013*a* ▸) and 6-(4-meth­oxy­phen­yl)-6a-nitro-6,6a,6 b,7,8,9,10,12a-octa­hydro­spiro­[chromeno[3,4-*a*]indolizine-12,3-indolin]-2-one (FIDCOM; Devi *et al.*, 2013*b* ▸). In both compounds, the piperidine ring has a chair conformation, as do the title compounds. In AFONEQ the pyran ring has a envelope conformation, as do molecules *A* and *B* of the title compounds, while in FIDCOM the pyran ring has a planar conformation. In these two compounds, the pyrrolidine ring adopts an envelope conformation as in mol­ecule A of the title compound. The bond lengths and bond angles are very similar to those reported here for the title compounds.

## Synthesis and crystallization   

To a solution of indeno­quinoxalinone (1.0 mmol) and pipacolinic acid (1.5 mmol) in dry toluene, was added 2-(4-chloro­phen­yl)-3-nitro-2*H*-chromene (1 mmol) under a nitro­gen atmosphere. The solution was refluxed for 20 h in a Dean–Stark apparatus to give the corresponding cyclo­adduct. After completion of the reaction, as indicated by TLC, the solvent was evaporated under reduced pressure. The crude product obtained was purified by column chromatography using hexa­ne/EtOAc (6:4) as eluent (yield 86%). Colourless block-like crystals of the title compound were obtained by slow evaporation of a solution in ethanol.

## Refinement   

Crystal data, data collection and structure refinement details are summarized in Table 2 ▸. All H atoms were positioned geometrically and constrained to ride on their parent atoms: C—H = 0.93–0.98 Å with *U*_iso_(H) = 1.5*U*_eq_(C-meth­yl) and 1.2*U*_eq_(C) for other H atoms.

In both mol­ecules, the nitro group oxygen atoms O3*A* and O4*A* in *A* and O3*B* and O4*B* in *B* are disordered over two positions with refined occupancy ratios of O3*A*/O4*A*:O3*A*′/O4*A*′ = 0.59 (2):0.41 (2), and O3*B*/O4*B*:O3*B*′/O4*B*′ = 0.686 (13):0.314 (13). In mol­ecule *B*, the chlorine atom Cl2 is disordered over two positions with a refined occupancy ratio of Cl2:Cl2′ = 0.72 (3):0.28 (3).

## Supplementary Material

Crystal structure: contains datablock(s) global, I. DOI: 10.1107/S2056989019000975/su5475sup1.cif

Structure factors: contains datablock(s) I. DOI: 10.1107/S2056989019000975/su5475Isup2.hkl

Supporting information file. DOI: 10.1107/S2056989019000975/su5475Isup3.cml

CCDC reference: 1024832

Additional supporting information:  crystallographic information; 3D view; checkCIF report

## Figures and Tables

**Figure 2:**
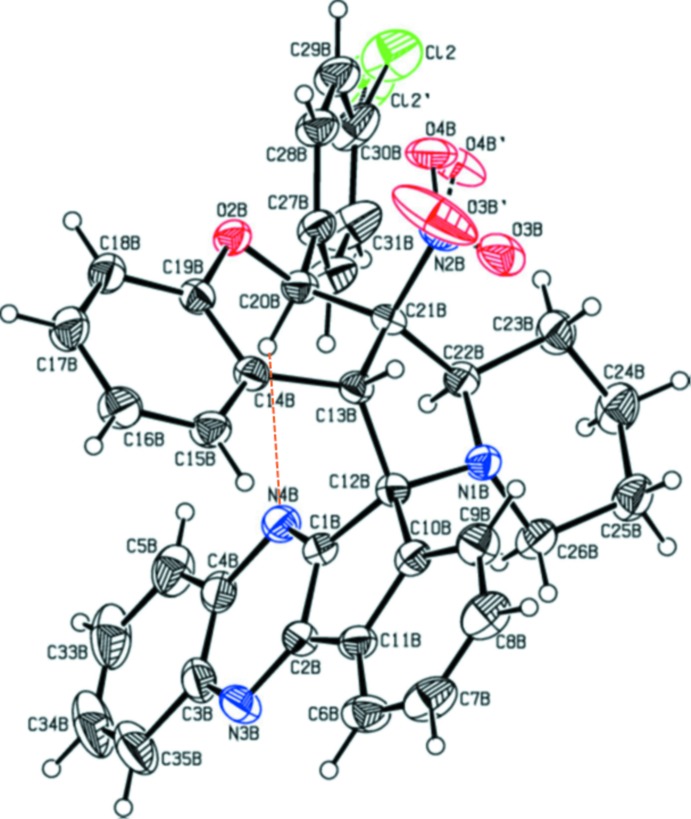
View of the mol­ecular structure of mol­ecule B of the title compound, with the atom labelling. Displacement ellipsoids are drawn at the 30% probability level. The intra­molecular C—H⋯N contact (Table 1 ▸) is shown as an orange dashed line.

**Table 1:** Hydrogen-bond geometry (Å, °) *Cg*1, *Cg*2 and *Cg*3 are the centroids of rings C14*B*–C19*B*, C3*B*–C5*B*/C33*B*–C35*B* and C3*A*–C5*A*/C33*A*–C35*A*, respectively.

*D*—H⋯*A*	*D*—H	H⋯*A*	*D*⋯*A*	*D*—H⋯*A*
C20*A*—H20*A*⋯N4*A*	0.98	2.28	3.170 (3)	151
C20*B*—H20*B*⋯N4*B*	0.98	2.45	3.298 (3)	144
C33*B*—H33*B*⋯O3*B* ^i^	0.93	2.46	3.271 (5)	145
C8*A*—H8*A*⋯*Cg*1^ii^	0.93	2.69	3.536 (3)	151
C25*B*—H25*C*⋯*Cg*2^iii^	0.97	2.73	3.629 (3)	155
C31*B*—H31*B*⋯*Cg*3^iv^	0.93	2.99	3.742 (3)	139

Symmetry codes: (i) 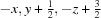; (ii) 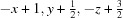; (iii) 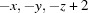; (iv) 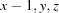.

**Table 2:** Experimental details

Crystal data	
Chemical formula	C_35_H_27_ClN_4_O_3_
*M*_r_	587.05
Crystal system, space group	Monoclinic, *P*2_1_/*c*
Temperature (K)	293
*a*, *b*, *c* (Å)	14.1880 (1), 23.4450 (3), 18.3710 (2)
β (°)	104.541 (2)
*V* (Å^3^)	5915.14 (12)
*Z*	8
Radiation type	Mo *K*α
μ (mm^−1^)	0.17
Crystal size (mm)	0.28 × 0.24 × 0.20

Data collection	
Diffractometer	Bruker Kappa APEXII CCD
Absorption correction	Multi-scan (*SADABS*; Bruker, 2008 ▸)
*T*_min_, *T*_max_	0.763, 0.841
No. of measured, independent and observed [*I* > 2σ(*I*)] reflections	47802, 11007, 6368
*R*_int_	0.049
(sin θ/λ)_max_ (Å^−1^)	0.606

Refinement	
*R*[*F* ^2^ > 2σ(*F* ^2^)], *wR*(*F* ^2^), *S*	0.048, 0.128, 1.04
No. of reflections	11007
No. of parameters	824
H-atom treatment	H-atom parameters constrained
Δρ_max_, Δρ_min_ (e Å^−3^)	0.35, −0.32

Computer programs: *APEX2* and *SAINT* (Bruker, 2008 ▸), *SHELXS2018* (Sheldrick, 2008 ▸), *SHELXL2018* (Sheldrick, 2015 ▸), *ORTEP-3 for Windows* and *WinGX* (Farrugia, 2012 ▸), *Mercury* (Macrae *et al.*, 2008 ▸), *publCIF* (Westrip, 2010 ▸) and *PLATON* (Spek, 2009 ▸).

## supplementary crystallographic information

### Crystal data

**Table:**

C_35_H_27_ClN_4_O_3_	*F*(000) = 2448
*M**_r_* = 587.05	*D*_x_ = 1.318 Mg m^−^^3^
Monoclinic, *P*2_1_/*c*	Mo *K*α radiation, λ = 0.71073 Å
*a* = 14.1880 (1) Å	Cell parameters from 14290 reflections
*b* = 23.4450 (3) Å	θ = 1.4–28.3°
*c* = 18.3710 (2) Å	µ = 0.17 mm^−^^1^
β = 104.541 (2)°	*T* = 293 K
*V* = 5915.14 (12) Å^3^	Block, colourless
*Z* = 8	0.28 × 0.24 × 0.20 mm

### Data collection

**Table:**

Bruker Kappa APEXII CCD diffractometer	6368 reflections with *I* > 2σ(*I*)
ω and φ scans	*R*_int_ = 0.049
Absorption correction: multi-scan (*SADABS*; Bruker, 2008)	θ_max_ = 25.5°, θ_min_ = 1.4°
*T*_min_ = 0.763, *T*_max_ = 0.841	*h* = −17→16
47802 measured reflections	*k* = −28→28
11007 independent reflections	*l* = −22→22

### Refinement

**Table:**

Refinement on *F*^2^	Secondary atom site location: difference Fourier map
Least-squares matrix: full	Hydrogen site location: inferred from neighbouring sites
*R*[*F*^2^ > 2σ(*F*^2^)] = 0.048	H-atom parameters constrained
*wR*(*F*^2^) = 0.128	*w* = 1/[σ^2^(*F*_o_^2^) + (0.0582*P*)^2^] where *P* = (*F*_o_^2^ + 2*F*_c_^2^)/3
*S* = 1.03	(Δ/σ)_max_ = 0.006
11007 reflections	Δρ_max_ = 0.35 e Å^−^^3^
824 parameters	Δρ_min_ = −0.32 e Å^−^^3^
0 restraints	Extinction correction: SHELXL2018 (Sheldrick, 2015), Fc^*^=kFc[1+0.001xFc^2^λ^3^/sin(2θ)]^-1/4^
Primary atom site location: structure-invariant direct methods	Extinction coefficient: 0.00059 (14)

### Special details

**Table:**

Geometry. All esds (except the esd in the dihedral angle between two l.s. planes) are estimated using the full covariance matrix. The cell esds are taken into account individually in the estimation of esds in distances, angles and torsion angles; correlations between esds in cell parameters are only used when they are defined by crystal symmetry. An approximate (isotropic) treatment of cell esds is used for estimating esds involving l.s. planes.

### Fractional atomic coordinates and isotropic or equivalent isotropic displacement parameters (Å^2^)

**Table:**

	*x*	*y*	*z*	*U*_iso_*/*U*_eq_	Occ. (<1)
Cl1A	−0.00824 (5)	0.11344 (4)	0.58679 (6)	0.1082 (3)	
O3A	0.4634 (4)	0.3265 (3)	0.6462 (7)	0.093 (3)	0.59 (2)
O4A	0.3489 (13)	0.2767 (4)	0.5822 (8)	0.156 (5)	0.59 (2)
O3A'	0.4590 (5)	0.2989 (9)	0.5979 (10)	0.109 (6)	0.41 (2)
O4A'	0.3204 (7)	0.2772 (5)	0.6066 (6)	0.071 (3)	0.41 (2)
O2A	0.45596 (11)	0.16374 (7)	0.58900 (9)	0.0664 (4)	
N1A	0.50758 (12)	0.25630 (7)	0.81689 (10)	0.0495 (5)	
N2A	0.4135 (2)	0.27834 (10)	0.63100 (16)	0.0724 (7)	
N3A	0.73468 (12)	0.10164 (7)	0.88894 (11)	0.0546 (5)	
N4A	0.53615 (12)	0.12159 (7)	0.80912 (10)	0.0479 (4)	
C1A	0.60146 (14)	0.16158 (8)	0.82389 (12)	0.0441 (5)	
C2A	0.69943 (14)	0.15201 (9)	0.86567 (13)	0.0491 (5)	
C3A	0.66770 (15)	0.05800 (8)	0.87139 (12)	0.0473 (5)	
C4A	0.56935 (15)	0.06792 (8)	0.83442 (12)	0.0464 (5)	
C5A	0.50372 (16)	0.02191 (9)	0.82077 (14)	0.0580 (6)	
H5A	0.438395	0.028115	0.797377	0.070*	
C6A	0.84079 (17)	0.22040 (11)	0.92049 (17)	0.0791 (8)	
H6A	0.883968	0.192052	0.943431	0.095*	
C7A	0.8676 (2)	0.27716 (12)	0.92813 (19)	0.0902 (9)	
H7A	0.929121	0.287229	0.956886	0.108*	
C8A	0.8040 (2)	0.31873 (11)	0.89352 (17)	0.0792 (8)	
H8A	0.822850	0.356768	0.899789	0.095*	
C9A	0.71276 (18)	0.30543 (10)	0.84969 (16)	0.0698 (7)	
H9A	0.670705	0.333954	0.825732	0.084*	
C10A	0.68507 (15)	0.24874 (9)	0.84208 (14)	0.0541 (6)	
C11A	0.74920 (15)	0.20657 (9)	0.87840 (14)	0.0556 (6)	
C12A	0.58742 (14)	0.22441 (8)	0.79981 (13)	0.0475 (5)	
C13A	0.56278 (15)	0.23380 (8)	0.71304 (13)	0.0501 (6)	
H13A	0.581056	0.273216	0.705252	0.060*	
C14A	0.61130 (16)	0.19680 (9)	0.66583 (14)	0.0555 (6)	
C15A	0.71259 (17)	0.19720 (11)	0.67750 (16)	0.0753 (8)	
H15A	0.750681	0.217889	0.717366	0.090*	
C16A	0.7565 (2)	0.16720 (14)	0.6304 (2)	0.0964 (10)	
H16A	0.823903	0.167393	0.638981	0.116*	
C17A	0.7008 (3)	0.13700 (15)	0.5708 (2)	0.1035 (11)	
H17A	0.730750	0.117208	0.538892	0.124*	
C18A	0.6011 (2)	0.13591 (12)	0.55817 (16)	0.0837 (8)	
H18A	0.563482	0.115436	0.517886	0.100*	
C19A	0.55733 (18)	0.16564 (10)	0.60611 (14)	0.0623 (6)	
C20A	0.41794 (15)	0.17284 (9)	0.65322 (12)	0.0528 (6)	
H20A	0.449631	0.144457	0.690557	0.063*	
C21A	0.45075 (15)	0.23143 (8)	0.68847 (12)	0.0484 (5)	
C22A	0.41783 (15)	0.24203 (9)	0.76128 (13)	0.0488 (5)	
H22A	0.394694	0.205552	0.776279	0.059*	
C23A	0.34060 (17)	0.28633 (10)	0.76270 (15)	0.0691 (7)	
H23A	0.279773	0.275799	0.727673	0.083*	
H23B	0.360646	0.323168	0.747801	0.083*	
C24A	0.32669 (19)	0.28976 (12)	0.84187 (17)	0.0853 (8)	
H24A	0.282177	0.320671	0.844364	0.102*	
H24B	0.297454	0.254555	0.853285	0.102*	
C25A	0.42135 (19)	0.29947 (12)	0.90013 (16)	0.0799 (8)	
H25A	0.409834	0.297599	0.949940	0.096*	
H25B	0.445771	0.337265	0.893556	0.096*	
C26A	0.49693 (18)	0.25542 (10)	0.89383 (14)	0.0655 (7)	
H26A	0.558686	0.264254	0.928826	0.079*	
H26B	0.476419	0.217866	0.905839	0.079*	
C27A	0.31104 (16)	0.15833 (9)	0.63324 (13)	0.0540 (6)	
C28A	0.24932 (17)	0.16813 (10)	0.56282 (14)	0.0660 (7)	
H28A	0.273935	0.183682	0.524812	0.079*	
C29A	0.15004 (18)	0.15474 (11)	0.54862 (15)	0.0733 (7)	
H29A	0.108512	0.161832	0.501616	0.088*	
C30A	0.11461 (17)	0.13090 (11)	0.60519 (17)	0.0694 (7)	
C31A	0.17473 (19)	0.11935 (11)	0.67385 (16)	0.0712 (7)	
H31A	0.150240	0.102750	0.711277	0.085*	
C32A	0.27203 (18)	0.13245 (10)	0.68733 (14)	0.0639 (6)	
H32A	0.313046	0.123757	0.734010	0.077*	
C33A	0.53600 (19)	−0.03179 (10)	0.84187 (15)	0.0684 (7)	
H33A	0.492516	−0.062175	0.831999	0.082*	
C34A	0.6328 (2)	−0.04175 (10)	0.87785 (15)	0.0694 (7)	
H34A	0.653630	−0.078651	0.891996	0.083*	
C35A	0.69781 (18)	0.00240 (9)	0.89266 (14)	0.0611 (6)	
H35A	0.762530	−0.004646	0.917074	0.073*	
Cl2	−0.4783 (4)	−0.0038 (8)	0.6049 (3)	0.1272 (17)	0.72 (3)
Cl2'	−0.4841 (8)	−0.0219 (5)	0.6031 (6)	0.075 (3)	0.28 (3)
O2B	−0.00811 (9)	−0.04381 (5)	0.61628 (8)	0.0465 (4)	
O3B	0.0158 (3)	−0.19290 (12)	0.7246 (4)	0.0803 (19)	0.686 (13)
O4B	−0.0989 (6)	−0.1554 (3)	0.6417 (4)	0.0762 (18)	0.686 (13)
O3B'	0.0164 (6)	−0.1739 (6)	0.6662 (11)	0.103 (7)	0.314 (13)
O4B'	−0.1267 (10)	−0.1518 (7)	0.6652 (10)	0.085 (4)	0.314 (13)
N1B	0.05316 (11)	−0.10204 (6)	0.86520 (9)	0.0425 (4)	
N2B	−0.03373 (16)	−0.14979 (8)	0.69290 (14)	0.0569 (5)	
N3B	0.26320 (15)	0.05649 (8)	0.92353 (11)	0.0627 (5)	
N4B	0.07324 (13)	0.02914 (7)	0.83140 (10)	0.0499 (5)	
C1B	0.14136 (15)	−0.00887 (8)	0.85633 (11)	0.0413 (5)	
C2B	0.23529 (15)	0.00447 (9)	0.90188 (12)	0.0469 (5)	
C3B	0.1926 (2)	0.09760 (9)	0.89932 (15)	0.0615 (7)	
C4B	0.09943 (19)	0.08441 (9)	0.85415 (13)	0.0551 (6)	
C5B	0.0311 (2)	0.12806 (10)	0.82974 (15)	0.0730 (8)	
H5B	−0.030148	0.119622	0.799127	0.088*	
C6B	0.38409 (16)	−0.05878 (12)	0.96269 (14)	0.0638 (7)	
H6B	0.424242	−0.028847	0.984698	0.077*	
C7B	0.41665 (17)	−0.11435 (13)	0.97233 (15)	0.0722 (7)	
H7B	0.479521	−0.121910	1.000663	0.087*	
C8B	0.35666 (18)	−0.15897 (11)	0.94023 (15)	0.0683 (7)	
H8B	0.379491	−0.196242	0.947687	0.082*	
C9B	0.26356 (16)	−0.14892 (9)	0.89737 (13)	0.0568 (6)	
H9B	0.223331	−0.179045	0.876142	0.068*	
C10B	0.23102 (14)	−0.09319 (8)	0.88649 (12)	0.0427 (5)	
C11B	0.29053 (14)	−0.04826 (9)	0.91966 (12)	0.0468 (5)	
C12B	0.13200 (13)	−0.07268 (7)	0.84181 (11)	0.0389 (5)	
C13B	0.10657 (13)	−0.08981 (7)	0.75708 (11)	0.0376 (5)	
H13B	0.129220	−0.129182	0.755638	0.045*	
C14B	0.14984 (14)	−0.05683 (8)	0.70283 (12)	0.0400 (5)	
C15B	0.25031 (15)	−0.05025 (9)	0.71576 (13)	0.0509 (5)	
H15B	0.290440	−0.061195	0.761853	0.061*	
C16B	0.29121 (16)	−0.02786 (10)	0.66161 (14)	0.0608 (6)	
H16B	0.358347	−0.023378	0.671331	0.073*	
C17B	0.23213 (16)	−0.01207 (10)	0.59272 (14)	0.0619 (6)	
H17B	0.259923	0.002197	0.555686	0.074*	
C18B	0.13295 (15)	−0.01723 (9)	0.57845 (13)	0.0530 (6)	
H18B	0.093362	−0.006287	0.532149	0.064*	
C19B	0.09235 (14)	−0.03897 (8)	0.63384 (12)	0.0414 (5)	
C20B	−0.04584 (14)	−0.04205 (8)	0.68166 (11)	0.0417 (5)	
H20B	−0.018549	−0.007621	0.709294	0.050*	
C21B	−0.00534 (13)	−0.09262 (7)	0.73357 (11)	0.0388 (5)	
C22B	−0.03703 (13)	−0.09156 (8)	0.80785 (12)	0.0427 (5)	
H22B	−0.057926	−0.052574	0.815036	0.051*	
C23B	−0.11678 (15)	−0.13179 (10)	0.81870 (14)	0.0601 (6)	
H23C	−0.177409	−0.123216	0.782169	0.072*	
H23D	−0.099102	−0.170920	0.811213	0.072*	
C24B	−0.12967 (17)	−0.12448 (11)	0.89788 (15)	0.0712 (7)	
H24C	−0.156099	−0.086913	0.902717	0.085*	
H24D	−0.175717	−0.152580	0.906753	0.085*	
C25B	−0.03347 (16)	−0.13142 (11)	0.95634 (14)	0.0666 (7)	
H25C	−0.042950	−0.123592	1.005857	0.080*	
H25D	−0.011283	−0.170526	0.955883	0.080*	
C26B	0.04359 (16)	−0.09150 (9)	0.94120 (13)	0.0558 (6)	
H26C	0.024780	−0.052179	0.946009	0.067*	
H26D	0.105333	−0.098277	0.977321	0.067*	
C27B	−0.15509 (14)	−0.03312 (9)	0.65835 (12)	0.0463 (5)	
C28B	−0.21425 (15)	−0.05551 (9)	0.59349 (13)	0.0542 (6)	
H28B	−0.186285	−0.075848	0.560808	0.065*	
C29B	−0.31419 (16)	−0.04849 (11)	0.57578 (14)	0.0657 (7)	
H29B	−0.353288	−0.064274	0.532108	0.079*	
C30B	−0.35442 (16)	−0.01785 (12)	0.62382 (15)	0.0712 (8)	
C31B	−0.29829 (18)	0.00675 (13)	0.68675 (16)	0.0824 (9)	
H31B	−0.326700	0.028081	0.718270	0.099*	
C32B	−0.19809 (17)	−0.00026 (10)	0.70355 (14)	0.0661 (7)	
H32B	−0.159238	0.017412	0.745877	0.079*	
C33B	0.0553 (3)	0.18321 (11)	0.8514 (2)	0.0940 (10)	
H33B	0.010335	0.212283	0.835047	0.113*	
C34B	0.1459 (3)	0.19590 (12)	0.8972 (2)	0.1033 (13)	
H34B	0.160509	0.233535	0.911693	0.124*	
C35B	0.2144 (2)	0.15477 (12)	0.92177 (18)	0.0885 (10)	
H35B	0.274860	0.164220	0.952918	0.106*	

### Atomic displacement parameters (Å^2^)

**Table:**

	*U*^11^	*U*^22^	*U*^33^	*U*^12^	*U*^13^	*U*^23^
Cl1A	0.0558 (4)	0.1295 (7)	0.1340 (8)	−0.0009 (4)	0.0141 (5)	−0.0236 (6)
O3A	0.090 (3)	0.060 (3)	0.125 (7)	0.012 (2)	0.019 (3)	0.041 (3)
O4A	0.211 (12)	0.103 (4)	0.096 (7)	−0.009 (6)	−0.069 (7)	0.044 (5)
O3A'	0.071 (4)	0.138 (11)	0.119 (10)	0.007 (4)	0.026 (5)	0.083 (9)
O4A'	0.050 (4)	0.089 (4)	0.068 (6)	0.030 (3)	0.000 (3)	0.036 (4)
O2A	0.0599 (10)	0.0933 (12)	0.0451 (10)	0.0200 (9)	0.0114 (8)	−0.0011 (8)
N1A	0.0466 (10)	0.0523 (10)	0.0482 (12)	0.0092 (8)	0.0096 (10)	0.0035 (9)
N2A	0.0817 (18)	0.0717 (17)	0.0695 (19)	0.0311 (14)	0.0297 (17)	0.0288 (15)
N3A	0.0496 (11)	0.0496 (11)	0.0604 (13)	0.0099 (9)	0.0060 (10)	0.0068 (9)
N4A	0.0468 (10)	0.0476 (10)	0.0464 (12)	0.0015 (9)	0.0064 (9)	0.0083 (8)
C1A	0.0420 (12)	0.0467 (12)	0.0429 (13)	0.0046 (10)	0.0092 (10)	0.0060 (10)
C2A	0.0415 (12)	0.0505 (13)	0.0524 (15)	0.0045 (10)	0.0064 (11)	0.0051 (11)
C3A	0.0532 (13)	0.0447 (12)	0.0432 (14)	0.0050 (11)	0.0110 (11)	0.0024 (10)
C4A	0.0538 (13)	0.0448 (12)	0.0400 (13)	0.0053 (11)	0.0105 (11)	0.0045 (10)
C5A	0.0574 (14)	0.0490 (14)	0.0625 (17)	−0.0005 (11)	0.0053 (12)	0.0059 (11)
C6A	0.0535 (16)	0.0696 (17)	0.103 (2)	−0.0053 (13)	−0.0017 (16)	0.0133 (15)
C7A	0.0613 (17)	0.084 (2)	0.111 (3)	−0.0188 (16)	−0.0050 (17)	0.0029 (18)
C8A	0.0722 (18)	0.0607 (16)	0.101 (2)	−0.0163 (15)	0.0157 (18)	0.0041 (16)
C9A	0.0645 (16)	0.0510 (15)	0.092 (2)	−0.0031 (12)	0.0161 (15)	0.0096 (13)
C10A	0.0499 (13)	0.0484 (13)	0.0635 (17)	0.0002 (11)	0.0131 (12)	0.0048 (11)
C11A	0.0445 (13)	0.0564 (14)	0.0624 (17)	−0.0016 (11)	0.0071 (12)	0.0029 (12)
C12A	0.0450 (12)	0.0421 (12)	0.0540 (15)	0.0075 (10)	0.0096 (11)	0.0083 (10)
C13A	0.0513 (13)	0.0471 (12)	0.0532 (15)	0.0067 (10)	0.0157 (12)	0.0123 (10)
C14A	0.0541 (14)	0.0605 (14)	0.0557 (17)	0.0138 (11)	0.0207 (13)	0.0146 (12)
C15A	0.0587 (16)	0.0902 (18)	0.085 (2)	0.0135 (14)	0.0327 (16)	0.0109 (15)
C16A	0.0688 (19)	0.125 (3)	0.106 (3)	0.0219 (18)	0.041 (2)	−0.002 (2)
C17A	0.097 (3)	0.132 (3)	0.098 (3)	0.038 (2)	0.055 (2)	−0.003 (2)
C18A	0.083 (2)	0.107 (2)	0.067 (2)	0.0278 (16)	0.0297 (17)	−0.0042 (16)
C19A	0.0624 (16)	0.0752 (16)	0.0528 (17)	0.0194 (13)	0.0207 (14)	0.0121 (13)
C20A	0.0532 (13)	0.0664 (14)	0.0379 (14)	0.0171 (11)	0.0100 (11)	0.0078 (11)
C21A	0.0503 (13)	0.0488 (12)	0.0462 (14)	0.0164 (10)	0.0124 (11)	0.0151 (10)
C22A	0.0491 (13)	0.0487 (12)	0.0490 (15)	0.0058 (10)	0.0133 (12)	0.0044 (10)
C23A	0.0566 (15)	0.0798 (16)	0.0704 (19)	0.0256 (13)	0.0151 (14)	−0.0013 (14)
C24A	0.0693 (18)	0.111 (2)	0.081 (2)	0.0211 (16)	0.0309 (17)	−0.0120 (17)
C25A	0.0826 (19)	0.098 (2)	0.0613 (19)	0.0209 (16)	0.0223 (16)	−0.0129 (15)
C26A	0.0728 (16)	0.0730 (16)	0.0498 (16)	0.0088 (13)	0.0139 (14)	0.0032 (12)
C27A	0.0527 (13)	0.0606 (14)	0.0439 (15)	0.0150 (11)	0.0036 (12)	−0.0028 (11)
C28A	0.0611 (16)	0.0875 (18)	0.0461 (16)	0.0169 (13)	0.0073 (13)	0.0003 (13)
C29A	0.0555 (16)	0.102 (2)	0.0532 (17)	0.0227 (14)	−0.0031 (14)	−0.0085 (15)
C30A	0.0519 (15)	0.0826 (17)	0.071 (2)	0.0077 (13)	0.0111 (15)	−0.0173 (15)
C31A	0.0637 (17)	0.0844 (18)	0.064 (2)	−0.0125 (14)	0.0140 (15)	−0.0043 (14)
C32A	0.0682 (17)	0.0716 (16)	0.0462 (16)	−0.0012 (13)	0.0036 (13)	0.0050 (12)
C33A	0.0770 (18)	0.0504 (14)	0.075 (2)	−0.0092 (13)	0.0149 (15)	0.0022 (13)
C34A	0.0859 (19)	0.0449 (14)	0.077 (2)	0.0088 (14)	0.0191 (16)	0.0082 (13)
C35A	0.0670 (15)	0.0500 (14)	0.0633 (17)	0.0163 (12)	0.0104 (13)	0.0062 (12)
Cl2	0.0427 (12)	0.233 (6)	0.107 (2)	0.020 (2)	0.0209 (14)	0.054 (2)
Cl2'	0.034 (2)	0.125 (6)	0.069 (4)	−0.001 (2)	0.018 (2)	−0.016 (5)
O2B	0.0380 (8)	0.0614 (9)	0.0388 (9)	−0.0054 (6)	0.0073 (7)	0.0037 (7)
O3B	0.087 (2)	0.0405 (17)	0.096 (4)	0.0010 (14)	−0.010 (2)	−0.0061 (18)
O4B	0.090 (5)	0.061 (2)	0.056 (3)	−0.010 (3)	−0.023 (3)	−0.011 (2)
O3B'	0.065 (5)	0.095 (7)	0.158 (15)	−0.025 (4)	0.047 (6)	−0.080 (9)
O4B'	0.056 (6)	0.077 (5)	0.113 (11)	−0.023 (4)	0.005 (5)	−0.039 (6)
N1B	0.0381 (9)	0.0496 (10)	0.0382 (11)	−0.0030 (8)	0.0066 (8)	0.0028 (8)
N2B	0.0561 (13)	0.0554 (13)	0.0594 (16)	−0.0195 (11)	0.0150 (14)	−0.0131 (12)
N3B	0.0740 (13)	0.0585 (12)	0.0564 (14)	−0.0248 (11)	0.0179 (11)	−0.0137 (10)
N4B	0.0648 (12)	0.0390 (10)	0.0437 (11)	0.0004 (9)	0.0094 (9)	−0.0022 (8)
C1B	0.0486 (12)	0.0422 (12)	0.0322 (12)	−0.0035 (10)	0.0086 (10)	−0.0009 (9)
C2B	0.0516 (13)	0.0487 (13)	0.0407 (14)	−0.0128 (11)	0.0119 (11)	−0.0033 (10)
C3B	0.0920 (19)	0.0442 (13)	0.0571 (17)	−0.0184 (14)	0.0350 (15)	−0.0052 (12)
C4B	0.0858 (18)	0.0403 (13)	0.0433 (15)	−0.0034 (13)	0.0238 (14)	0.0010 (10)
C5B	0.109 (2)	0.0484 (15)	0.0691 (19)	0.0136 (14)	0.0356 (17)	0.0049 (13)
C6B	0.0423 (13)	0.0922 (19)	0.0542 (17)	−0.0118 (13)	0.0069 (12)	−0.0103 (14)
C7B	0.0450 (14)	0.111 (2)	0.0561 (18)	0.0170 (15)	0.0036 (13)	0.0054 (16)
C8B	0.0595 (16)	0.0815 (18)	0.0620 (18)	0.0246 (14)	0.0113 (14)	0.0097 (14)
C9B	0.0527 (14)	0.0566 (14)	0.0586 (16)	0.0065 (11)	0.0092 (13)	0.0033 (11)
C10B	0.0411 (11)	0.0487 (12)	0.0378 (13)	0.0019 (10)	0.0090 (10)	0.0036 (10)
C11B	0.0404 (12)	0.0609 (14)	0.0371 (13)	−0.0075 (11)	0.0060 (10)	−0.0014 (10)
C12B	0.0397 (11)	0.0380 (11)	0.0372 (13)	−0.0016 (9)	0.0062 (10)	0.0001 (9)
C13B	0.0385 (11)	0.0347 (10)	0.0381 (12)	−0.0007 (8)	0.0069 (10)	−0.0025 (9)
C14B	0.0400 (11)	0.0401 (11)	0.0406 (13)	−0.0030 (9)	0.0118 (10)	−0.0039 (9)
C15B	0.0413 (12)	0.0654 (14)	0.0447 (14)	−0.0050 (10)	0.0083 (11)	−0.0023 (11)
C16B	0.0409 (13)	0.0850 (17)	0.0582 (17)	−0.0112 (12)	0.0155 (13)	−0.0006 (13)
C17B	0.0555 (15)	0.0802 (16)	0.0545 (17)	−0.0104 (13)	0.0220 (13)	0.0077 (13)
C18B	0.0509 (13)	0.0595 (13)	0.0485 (15)	−0.0051 (11)	0.0123 (12)	0.0068 (11)
C19B	0.0384 (11)	0.0416 (11)	0.0444 (14)	−0.0027 (9)	0.0106 (11)	−0.0015 (9)
C20B	0.0398 (11)	0.0465 (12)	0.0368 (13)	−0.0049 (9)	0.0058 (10)	−0.0025 (9)
C21B	0.0391 (11)	0.0366 (10)	0.0383 (13)	−0.0083 (9)	0.0053 (10)	−0.0067 (9)
C22B	0.0399 (11)	0.0457 (11)	0.0417 (13)	−0.0029 (9)	0.0086 (10)	0.0009 (9)
C23B	0.0417 (12)	0.0777 (16)	0.0625 (17)	−0.0116 (11)	0.0160 (12)	0.0107 (13)
C24B	0.0505 (14)	0.0999 (19)	0.0679 (19)	0.0002 (13)	0.0235 (14)	0.0182 (15)
C25B	0.0590 (15)	0.0902 (18)	0.0534 (17)	−0.0021 (13)	0.0195 (13)	0.0159 (13)
C26B	0.0580 (14)	0.0680 (14)	0.0409 (14)	0.0015 (12)	0.0118 (12)	0.0039 (11)
C27B	0.0406 (12)	0.0606 (13)	0.0359 (13)	0.0009 (10)	0.0062 (11)	0.0054 (11)
C28B	0.0432 (13)	0.0741 (15)	0.0440 (15)	−0.0028 (11)	0.0083 (12)	−0.0002 (12)
C29B	0.0445 (13)	0.0996 (19)	0.0488 (16)	−0.0106 (13)	0.0039 (12)	0.0070 (14)
C30B	0.0362 (12)	0.128 (2)	0.0480 (17)	0.0101 (14)	0.0077 (13)	0.0243 (16)
C31B	0.0577 (16)	0.141 (3)	0.0496 (18)	0.0362 (16)	0.0158 (15)	0.0031 (17)
C32B	0.0585 (15)	0.0905 (17)	0.0443 (15)	0.0173 (13)	0.0036 (12)	−0.0043 (13)
C33B	0.151 (3)	0.0446 (17)	0.109 (3)	0.0099 (18)	0.074 (3)	0.0111 (16)
C34B	0.159 (3)	0.0409 (17)	0.140 (3)	−0.022 (2)	0.093 (3)	−0.0154 (19)
C35B	0.121 (2)	0.0559 (17)	0.103 (3)	−0.0356 (17)	0.055 (2)	−0.0220 (16)

### Geometric parameters (Å, º)

**Table:**

Cl1A—C30A	1.739 (2)	Cl2'—C30B	1.785 (11)
O3A—N2A	1.325 (8)	O2B—C19B	1.385 (2)
O4A—N2A	1.110 (9)	O2B—C20B	1.433 (2)
O3A'—N2A	1.102 (7)	O3B—N2B	1.284 (4)
O4A'—N2A	1.284 (12)	O4B—N2B	1.149 (6)
O2A—C19A	1.394 (3)	O3B'—N2B	1.115 (7)
O2A—C20A	1.431 (2)	O4B'—N2B	1.290 (15)
N1A—C12A	1.456 (2)	N1B—C26B	1.457 (3)
N1A—C22A	1.457 (3)	N1B—C22B	1.460 (2)
N1A—C26A	1.459 (3)	N1B—C12B	1.467 (2)
N2A—C21A	1.524 (3)	N2B—C21B	1.538 (3)
N3A—C2A	1.312 (2)	N3B—C2B	1.313 (2)
N3A—C3A	1.378 (3)	N3B—C3B	1.381 (3)
N4A—C1A	1.298 (2)	N4B—C1B	1.310 (2)
N4A—C4A	1.383 (2)	N4B—C4B	1.383 (3)
C1A—C2A	1.427 (3)	C1B—C2B	1.419 (3)
C1A—C12A	1.537 (3)	C1B—C12B	1.519 (3)
C2A—C11A	1.451 (3)	C2B—C11B	1.456 (3)
C3A—C35A	1.397 (3)	C3B—C4B	1.407 (3)
C3A—C4A	1.409 (3)	C3B—C35B	1.414 (3)
C4A—C5A	1.406 (3)	C4B—C5B	1.404 (3)
C5A—C33A	1.362 (3)	C5B—C33B	1.371 (4)
C5A—H5A	0.9300	C5B—H5B	0.9300
C6A—C11A	1.374 (3)	C6B—C7B	1.379 (3)
C6A—C7A	1.382 (3)	C6B—C11B	1.386 (3)
C6A—H6A	0.9300	C6B—H6B	0.9300
C7A—C8A	1.372 (4)	C7B—C8B	1.384 (3)
C7A—H7A	0.9300	C7B—H7B	0.9300
C8A—C9A	1.377 (3)	C8B—C9B	1.378 (3)
C8A—H8A	0.9300	C8B—H8B	0.9300
C9A—C10A	1.383 (3)	C9B—C10B	1.383 (3)
C9A—H9A	0.9300	C9B—H9B	0.9300
C10A—C11A	1.395 (3)	C10B—C11B	1.391 (3)
C10A—C12A	1.519 (3)	C10B—C12B	1.517 (3)
C12A—C13A	1.559 (3)	C12B—C13B	1.560 (3)
C13A—C14A	1.509 (3)	C13B—C14B	1.508 (3)
C13A—C21A	1.540 (3)	C13B—C21B	1.539 (2)
C13A—H13A	0.9800	C13B—H13B	0.9800
C14A—C19A	1.379 (3)	C14B—C19B	1.388 (3)
C14A—C15A	1.399 (3)	C14B—C15B	1.394 (3)
C15A—C16A	1.379 (4)	C15B—C16B	1.375 (3)
C15A—H15A	0.9300	C15B—H15B	0.9300
C16A—C17A	1.374 (4)	C16B—C17B	1.382 (3)
C16A—H16A	0.9300	C16B—H16B	0.9300
C17A—C18A	1.375 (4)	C17B—C18B	1.370 (3)
C17A—H17A	0.9300	C17B—H17B	0.9300
C18A—C19A	1.387 (3)	C18B—C19B	1.386 (3)
C18A—H18A	0.9300	C18B—H18B	0.9300
C20A—C27A	1.507 (3)	C20B—C27B	1.515 (3)
C20A—C21A	1.540 (3)	C20B—C21B	1.540 (3)
C20A—H20A	0.9800	C20B—H20B	0.9800
C21A—C22A	1.544 (3)	C21B—C22B	1.540 (3)
C22A—C23A	1.515 (3)	C22B—C23B	1.524 (3)
C22A—H22A	0.9800	C22B—H22B	0.9800
C23A—C24A	1.518 (4)	C23B—C24B	1.521 (3)
C23A—H23A	0.9700	C23B—H23C	0.9700
C23A—H23B	0.9700	C23B—H23D	0.9700
C24A—C25A	1.510 (4)	C24B—C25B	1.519 (3)
C24A—H24A	0.9700	C24B—H24C	0.9700
C24A—H24B	0.9700	C24B—H24D	0.9700
C25A—C26A	1.514 (3)	C25B—C26B	1.517 (3)
C25A—H25A	0.9700	C25B—H25C	0.9700
C25A—H25B	0.9700	C25B—H25D	0.9700
C26A—H26A	0.9700	C26B—H26C	0.9700
C26A—H26B	0.9700	C26B—H26D	0.9700
C27A—C28A	1.387 (3)	C27B—C28B	1.378 (3)
C27A—C32A	1.392 (3)	C27B—C32B	1.382 (3)
C28A—C29A	1.402 (3)	C28B—C29B	1.383 (3)
C28A—H28A	0.9300	C28B—H28B	0.9300
C29A—C30A	1.381 (4)	C29B—C30B	1.369 (3)
C29A—H29A	0.9300	C29B—H29B	0.9300
C30A—C31A	1.361 (4)	C30B—C31B	1.357 (4)
C31A—C32A	1.375 (3)	C31B—C32B	1.387 (3)
C31A—H31A	0.9300	C31B—H31B	0.9300
C32A—H32A	0.9300	C32B—H32B	0.9300
C33A—C34A	1.386 (3)	C33B—C34B	1.380 (5)
C33A—H33A	0.9300	C33B—H33B	0.9300
C34A—C35A	1.368 (3)	C34B—C35B	1.364 (4)
C34A—H34A	0.9300	C34B—H34B	0.9300
C35A—H35A	0.9300	C35B—H35B	0.9300
Cl2—C30B	1.735 (5)		
			
C19A—O2A—C20A	112.99 (17)	C26B—N1B—C22B	112.79 (15)
C12A—N1A—C22A	108.73 (16)	C26B—N1B—C12B	117.55 (16)
C12A—N1A—C26A	118.50 (18)	C22B—N1B—C12B	107.72 (15)
C22A—N1A—C26A	113.02 (17)	O4B—N2B—O3B	121.1 (4)
O3A'—N2A—O4A'	120.6 (6)	O3B'—N2B—O4B'	120.7 (7)
O4A—N2A—O3A	119.3 (6)	O3B'—N2B—C21B	122.5 (4)
O3A'—N2A—C21A	123.2 (5)	O4B—N2B—C21B	123.8 (4)
O4A—N2A—C21A	127.4 (6)	O3B—N2B—C21B	114.7 (2)
O4A'—N2A—C21A	112.0 (5)	O4B'—N2B—C21B	109.9 (7)
O3A—N2A—C21A	113.2 (4)	C2B—N3B—C3B	114.3 (2)
C2A—N3A—C3A	114.08 (18)	C1B—N4B—C4B	114.39 (19)
C1A—N4A—C4A	114.97 (17)	N4B—C1B—C2B	123.88 (18)
N4A—C1A—C2A	123.15 (18)	N4B—C1B—C12B	125.74 (18)
N4A—C1A—C12A	126.74 (18)	C2B—C1B—C12B	110.38 (17)
C2A—C1A—C12A	110.11 (17)	N3B—C2B—C1B	123.4 (2)
N3A—C2A—C1A	123.86 (19)	N3B—C2B—C11B	128.0 (2)
N3A—C2A—C11A	127.6 (2)	C1B—C2B—C11B	108.55 (17)
C1A—C2A—C11A	108.48 (18)	N3B—C3B—C4B	122.31 (19)
N3A—C3A—C35A	118.9 (2)	N3B—C3B—C35B	118.7 (3)
N3A—C3A—C4A	122.10 (18)	C4B—C3B—C35B	119.0 (3)
C35A—C3A—C4A	119.0 (2)	N4B—C4B—C5B	118.3 (2)
N4A—C4A—C5A	118.9 (2)	N4B—C4B—C3B	121.7 (2)
N4A—C4A—C3A	121.64 (18)	C5B—C4B—C3B	120.0 (2)
C5A—C4A—C3A	119.38 (19)	C33B—C5B—C4B	119.5 (3)
C33A—C5A—C4A	119.9 (2)	C33B—C5B—H5B	120.2
C33A—C5A—H5A	120.1	C4B—C5B—H5B	120.2
C4A—C5A—H5A	120.1	C7B—C6B—C11B	118.9 (2)
C11A—C6A—C7A	118.8 (2)	C7B—C6B—H6B	120.5
C11A—C6A—H6A	120.6	C11B—C6B—H6B	120.5
C7A—C6A—H6A	120.6	C6B—C7B—C8B	120.6 (2)
C8A—C7A—C6A	120.3 (3)	C6B—C7B—H7B	119.7
C8A—C7A—H7A	119.8	C8B—C7B—H7B	119.7
C6A—C7A—H7A	119.8	C9B—C8B—C7B	120.9 (2)
C7A—C8A—C9A	121.5 (2)	C9B—C8B—H8B	119.6
C7A—C8A—H8A	119.2	C7B—C8B—H8B	119.6
C9A—C8A—H8A	119.2	C8B—C9B—C10B	118.8 (2)
C8A—C9A—C10A	118.5 (2)	C8B—C9B—H9B	120.6
C8A—C9A—H9A	120.7	C10B—C9B—H9B	120.6
C10A—C9A—H9A	120.7	C9B—C10B—C11B	120.5 (2)
C9A—C10A—C11A	119.9 (2)	C9B—C10B—C12B	127.44 (19)
C9A—C10A—C12A	127.7 (2)	C11B—C10B—C12B	112.01 (17)
C11A—C10A—C12A	112.34 (18)	C6B—C11B—C10B	120.3 (2)
C6A—C11A—C10A	120.9 (2)	C6B—C11B—C2B	131.6 (2)
C6A—C11A—C2A	130.7 (2)	C10B—C11B—C2B	108.13 (18)
C10A—C11A—C2A	108.37 (19)	N1B—C12B—C10B	111.32 (15)
N1A—C12A—C10A	110.86 (17)	N1B—C12B—C1B	116.76 (15)
N1A—C12A—C1A	118.80 (16)	C10B—C12B—C1B	100.89 (15)
C10A—C12A—C1A	100.20 (16)	N1B—C12B—C13B	99.74 (14)
N1A—C12A—C13A	99.27 (16)	C10B—C12B—C13B	114.16 (15)
C10A—C12A—C13A	114.28 (17)	C1B—C12B—C13B	114.64 (15)
C1A—C12A—C13A	114.15 (17)	C14B—C13B—C21B	113.52 (16)
C14A—C13A—C21A	113.38 (19)	C14B—C13B—C12B	119.80 (15)
C14A—C13A—C12A	119.26 (17)	C21B—C13B—C12B	104.77 (14)
C21A—C13A—C12A	104.14 (16)	C14B—C13B—H13B	105.9
C14A—C13A—H13A	106.4	C21B—C13B—H13B	105.9
C21A—C13A—H13A	106.4	C12B—C13B—H13B	105.9
C12A—C13A—H13A	106.4	C19B—C14B—C15B	117.62 (18)
C19A—C14A—C15A	118.0 (2)	C19B—C14B—C13B	120.96 (17)
C19A—C14A—C13A	121.3 (2)	C15B—C14B—C13B	120.84 (18)
C15A—C14A—C13A	120.5 (2)	C16B—C15B—C14B	121.2 (2)
C16A—C15A—C14A	120.6 (3)	C16B—C15B—H15B	119.4
C16A—C15A—H15A	119.7	C14B—C15B—H15B	119.4
C14A—C15A—H15A	119.7	C15B—C16B—C17B	119.7 (2)
C17A—C16A—C15A	120.2 (3)	C15B—C16B—H16B	120.1
C17A—C16A—H16A	119.9	C17B—C16B—H16B	120.1
C15A—C16A—H16A	119.9	C18B—C17B—C16B	120.6 (2)
C18A—C17A—C16A	120.3 (3)	C18B—C17B—H17B	119.7
C18A—C17A—H17A	119.8	C16B—C17B—H17B	119.7
C16A—C17A—H17A	119.8	C17B—C18B—C19B	119.2 (2)
C17A—C18A—C19A	119.3 (3)	C17B—C18B—H18B	120.4
C17A—C18A—H18A	120.3	C19B—C18B—H18B	120.4
C19A—C18A—H18A	120.3	O2B—C19B—C18B	116.89 (19)
C14A—C19A—C18A	121.6 (2)	O2B—C19B—C14B	121.49 (17)
C14A—C19A—O2A	122.1 (2)	C18B—C19B—C14B	121.59 (18)
C18A—C19A—O2A	116.3 (2)	O2B—C20B—C27B	109.76 (16)
O2A—C20A—C27A	109.28 (18)	O2B—C20B—C21B	109.69 (14)
O2A—C20A—C21A	110.05 (17)	C27B—C20B—C21B	118.53 (15)
C27A—C20A—C21A	118.89 (17)	O2B—C20B—H20B	106.0
O2A—C20A—H20A	105.9	C27B—C20B—H20B	106.0
C27A—C20A—H20A	105.9	C21B—C20B—H20B	106.0
C21A—C20A—H20A	105.9	N2B—C21B—C13B	107.44 (15)
N2A—C21A—C20A	109.7 (2)	N2B—C21B—C20B	111.02 (17)
N2A—C21A—C13A	109.26 (19)	C13B—C21B—C20B	109.83 (14)
C20A—C21A—C13A	109.64 (16)	N2B—C21B—C22B	110.21 (15)
N2A—C21A—C22A	110.86 (17)	C13B—C21B—C22B	105.12 (16)
C20A—C21A—C22A	112.50 (16)	C20B—C21B—C22B	112.93 (15)
C13A—C21A—C22A	104.76 (18)	N1B—C22B—C23B	110.28 (17)
N1A—C22A—C23A	109.82 (18)	N1B—C22B—C21B	103.81 (14)
N1A—C22A—C21A	104.07 (16)	C23B—C22B—C21B	119.82 (17)
C23A—C22A—C21A	120.19 (18)	N1B—C22B—H22B	107.4
N1A—C22A—H22A	107.4	C23B—C22B—H22B	107.4
C23A—C22A—H22A	107.4	C21B—C22B—H22B	107.4
C21A—C22A—H22A	107.4	C24B—C23B—C22B	109.08 (19)
C22A—C23A—C24A	108.7 (2)	C24B—C23B—H23C	109.9
C22A—C23A—H23A	109.9	C22B—C23B—H23C	109.9
C24A—C23A—H23A	109.9	C24B—C23B—H23D	109.9
C22A—C23A—H23B	109.9	C22B—C23B—H23D	109.9
C24A—C23A—H23B	109.9	H23C—C23B—H23D	108.3
H23A—C23A—H23B	108.3	C25B—C24B—C23B	111.20 (18)
C25A—C24A—C23A	112.4 (2)	C25B—C24B—H24C	109.4
C25A—C24A—H24A	109.1	C23B—C24B—H24C	109.4
C23A—C24A—H24A	109.1	C25B—C24B—H24D	109.4
C25A—C24A—H24B	109.1	C23B—C24B—H24D	109.4
C23A—C24A—H24B	109.1	H24C—C24B—H24D	108.0
H24A—C24A—H24B	107.9	C26B—C25B—C24B	111.43 (19)
C24A—C25A—C26A	111.3 (2)	C26B—C25B—H25C	109.3
C24A—C25A—H25A	109.4	C24B—C25B—H25C	109.3
C26A—C25A—H25A	109.4	C26B—C25B—H25D	109.3
C24A—C25A—H25B	109.4	C24B—C25B—H25D	109.3
C26A—C25A—H25B	109.4	H25C—C25B—H25D	108.0
H25A—C25A—H25B	108.0	N1B—C26B—C25B	108.41 (18)
N1A—C26A—C25A	108.2 (2)	N1B—C26B—H26C	110.0
N1A—C26A—H26A	110.1	C25B—C26B—H26C	110.0
C25A—C26A—H26A	110.1	N1B—C26B—H26D	110.0
N1A—C26A—H26B	110.1	C25B—C26B—H26D	110.0
C25A—C26A—H26B	110.1	H26C—C26B—H26D	108.4
H26A—C26A—H26B	108.4	C28B—C27B—C32B	117.8 (2)
C28A—C27A—C32A	117.8 (2)	C28B—C27B—C20B	123.35 (18)
C28A—C27A—C20A	123.4 (2)	C32B—C27B—C20B	118.8 (2)
C32A—C27A—C20A	118.7 (2)	C27B—C28B—C29B	121.6 (2)
C27A—C28A—C29A	120.4 (2)	C27B—C28B—H28B	119.2
C27A—C28A—H28A	119.8	C29B—C28B—H28B	119.2
C29A—C28A—H28A	119.8	C30B—C29B—C28B	118.7 (2)
C30A—C29A—C28A	119.3 (2)	C30B—C29B—H29B	120.7
C30A—C29A—H29A	120.4	C28B—C29B—H29B	120.7
C28A—C29A—H29A	120.4	C31B—C30B—C29B	121.5 (2)
C31A—C30A—C29A	121.1 (2)	C31B—C30B—Cl2	116.0 (5)
C31A—C30A—Cl1A	119.8 (2)	C29B—C30B—Cl2	122.4 (4)
C29A—C30A—Cl1A	119.0 (2)	C31B—C30B—Cl2'	123.9 (3)
C30A—C31A—C32A	119.3 (2)	C29B—C30B—Cl2'	114.1 (3)
C30A—C31A—H31A	120.3	C30B—C31B—C32B	119.3 (2)
C32A—C31A—H31A	120.3	C30B—C31B—H31B	120.4
C31A—C32A—C27A	122.0 (2)	C32B—C31B—H31B	120.4
C31A—C32A—H32A	119.0	C27B—C32B—C31B	121.0 (2)
C27A—C32A—H32A	119.0	C27B—C32B—H32B	119.5
C5A—C33A—C34A	120.9 (2)	C31B—C32B—H32B	119.5
C5A—C33A—H33A	119.6	C5B—C33B—C34B	120.5 (3)
C34A—C33A—H33A	119.6	C5B—C33B—H33B	119.8
C35A—C34A—C33A	120.4 (2)	C34B—C33B—H33B	119.8
C35A—C34A—H34A	119.8	C35B—C34B—C33B	121.8 (3)
C33A—C34A—H34A	119.8	C35B—C34B—H34B	119.1
C34A—C35A—C3A	120.5 (2)	C33B—C34B—H34B	119.1
C34A—C35A—H35A	119.8	C34B—C35B—C3B	119.2 (3)
C3A—C35A—H35A	119.8	C34B—C35B—H35B	120.4
C19B—O2B—C20B	112.44 (15)	C3B—C35B—H35B	120.4
			
C4A—N4A—C1A—C2A	−2.5 (3)	C4B—N4B—C1B—C12B	178.48 (17)
C4A—N4A—C1A—C12A	176.95 (19)	C3B—N3B—C2B—C1B	1.1 (3)
C3A—N3A—C2A—C1A	−1.8 (3)	C3B—N3B—C2B—C11B	−177.9 (2)
C3A—N3A—C2A—C11A	176.6 (2)	N4B—C1B—C2B—N3B	0.0 (3)
N4A—C1A—C2A—N3A	4.6 (3)	C12B—C1B—C2B—N3B	−179.61 (18)
C12A—C1A—C2A—N3A	−174.9 (2)	N4B—C1B—C2B—C11B	179.10 (18)
N4A—C1A—C2A—C11A	−174.02 (19)	C12B—C1B—C2B—C11B	−0.5 (2)
C12A—C1A—C2A—C11A	6.5 (2)	C2B—N3B—C3B—C4B	−1.1 (3)
C2A—N3A—C3A—C35A	179.2 (2)	C2B—N3B—C3B—C35B	177.9 (2)
C2A—N3A—C3A—C4A	−2.5 (3)	C1B—N4B—C4B—C5B	179.66 (19)
C1A—N4A—C4A—C5A	−179.82 (19)	C1B—N4B—C4B—C3B	1.0 (3)
C1A—N4A—C4A—C3A	−1.8 (3)	N3B—C3B—C4B—N4B	0.1 (3)
N3A—C3A—C4A—N4A	4.5 (3)	C35B—C3B—C4B—N4B	−179.0 (2)
C35A—C3A—C4A—N4A	−177.15 (19)	N3B—C3B—C4B—C5B	−178.6 (2)
N3A—C3A—C4A—C5A	−177.5 (2)	C35B—C3B—C4B—C5B	2.4 (3)
C35A—C3A—C4A—C5A	0.9 (3)	N4B—C4B—C5B—C33B	−179.9 (2)
N4A—C4A—C5A—C33A	176.7 (2)	C3B—C4B—C5B—C33B	−1.2 (3)
C3A—C4A—C5A—C33A	−1.4 (3)	C11B—C6B—C7B—C8B	−0.6 (4)
C11A—C6A—C7A—C8A	−0.7 (5)	C6B—C7B—C8B—C9B	0.7 (4)
C6A—C7A—C8A—C9A	−0.9 (5)	C7B—C8B—C9B—C10B	0.4 (3)
C7A—C8A—C9A—C10A	1.2 (4)	C8B—C9B—C10B—C11B	−1.4 (3)
C8A—C9A—C10A—C11A	−0.1 (4)	C8B—C9B—C10B—C12B	−179.9 (2)
C8A—C9A—C10A—C12A	177.1 (2)	C7B—C6B—C11B—C10B	−0.4 (3)
C7A—C6A—C11A—C10A	1.8 (4)	C7B—C6B—C11B—C2B	177.4 (2)
C7A—C6A—C11A—C2A	−174.7 (3)	C9B—C10B—C11B—C6B	1.4 (3)
C9A—C10A—C11A—C6A	−1.5 (4)	C12B—C10B—C11B—C6B	−179.87 (19)
C12A—C10A—C11A—C6A	−179.1 (2)	C9B—C10B—C11B—C2B	−176.84 (18)
C9A—C10A—C11A—C2A	175.7 (2)	C12B—C10B—C11B—C2B	1.8 (2)
C12A—C10A—C11A—C2A	−1.9 (3)	N3B—C2B—C11B—C6B	0.2 (4)
N3A—C2A—C11A—C6A	−4.6 (4)	C1B—C2B—C11B—C6B	−178.9 (2)
C1A—C2A—C11A—C6A	173.9 (3)	N3B—C2B—C11B—C10B	178.2 (2)
N3A—C2A—C11A—C10A	178.6 (2)	C1B—C2B—C11B—C10B	−0.8 (2)
C1A—C2A—C11A—C10A	−2.9 (3)	C26B—N1B—C12B—C10B	66.0 (2)
C22A—N1A—C12A—C10A	164.91 (17)	C22B—N1B—C12B—C10B	−165.34 (15)
C26A—N1A—C12A—C10A	−64.2 (2)	C26B—N1B—C12B—C1B	−49.1 (2)
C22A—N1A—C12A—C1A	−79.9 (2)	C22B—N1B—C12B—C1B	79.6 (2)
C26A—N1A—C12A—C1A	51.0 (3)	C26B—N1B—C12B—C13B	−173.19 (16)
C22A—N1A—C12A—C13A	44.37 (19)	C22B—N1B—C12B—C13B	−44.49 (17)
C26A—N1A—C12A—C13A	175.23 (18)	C9B—C10B—C12B—N1B	52.0 (3)
C9A—C10A—C12A—N1A	−45.7 (3)	C11B—C10B—C12B—N1B	−126.56 (17)
C11A—C10A—C12A—N1A	131.70 (19)	C9B—C10B—C12B—C1B	176.56 (19)
C9A—C10A—C12A—C1A	−172.0 (2)	C11B—C10B—C12B—C1B	−2.0 (2)
C11A—C10A—C12A—C1A	5.4 (2)	C9B—C10B—C12B—C13B	−60.0 (3)
C9A—C10A—C12A—C13A	65.5 (3)	C11B—C10B—C12B—C13B	121.47 (18)
C11A—C10A—C12A—C13A	−117.1 (2)	N4B—C1B—C12B—N1B	−57.3 (3)
N4A—C1A—C12A—N1A	52.7 (3)	C2B—C1B—C12B—N1B	122.22 (18)
C2A—C1A—C12A—N1A	−127.8 (2)	N4B—C1B—C12B—C10B	−178.12 (18)
N4A—C1A—C12A—C10A	173.5 (2)	C2B—C1B—C12B—C10B	1.4 (2)
C2A—C1A—C12A—C10A	−7.0 (2)	N4B—C1B—C12B—C13B	58.7 (2)
N4A—C1A—C12A—C13A	−63.9 (3)	C2B—C1B—C12B—C13B	−121.69 (18)
C2A—C1A—C12A—C13A	115.5 (2)	N1B—C12B—C13B—C14B	162.78 (16)
N1A—C12A—C13A—C14A	−165.11 (18)	C10B—C12B—C13B—C14B	−78.4 (2)
C10A—C12A—C13A—C14A	76.9 (2)	C1B—C12B—C13B—C14B	37.2 (2)
C1A—C12A—C13A—C14A	−37.7 (3)	N1B—C12B—C13B—C21B	33.99 (17)
N1A—C12A—C13A—C21A	−37.50 (18)	C10B—C12B—C13B—C21B	152.76 (15)
C10A—C12A—C13A—C21A	−155.51 (16)	C1B—C12B—C13B—C21B	−91.55 (17)
C1A—C12A—C13A—C21A	89.96 (19)	C21B—C13B—C14B—C19B	−8.9 (2)
C21A—C13A—C14A—C19A	1.3 (3)	C12B—C13B—C14B—C19B	−133.61 (18)
C12A—C13A—C14A—C19A	124.5 (2)	C21B—C13B—C14B—C15B	−179.94 (17)
C21A—C13A—C14A—C15A	175.57 (19)	C12B—C13B—C14B—C15B	55.3 (2)
C12A—C13A—C14A—C15A	−61.2 (3)	C19B—C14B—C15B—C16B	−1.2 (3)
C19A—C14A—C15A—C16A	0.0 (4)	C13B—C14B—C15B—C16B	170.14 (19)
C13A—C14A—C15A—C16A	−174.5 (2)	C14B—C15B—C16B—C17B	−0.7 (3)
C14A—C15A—C16A—C17A	0.7 (5)	C15B—C16B—C17B—C18B	1.6 (4)
C15A—C16A—C17A—C18A	−0.8 (5)	C16B—C17B—C18B—C19B	−0.5 (3)
C16A—C17A—C18A—C19A	0.1 (5)	C20B—O2B—C19B—C18B	−154.42 (17)
C15A—C14A—C19A—C18A	−0.7 (4)	C20B—O2B—C19B—C14B	27.7 (2)
C13A—C14A—C19A—C18A	173.8 (2)	C17B—C18B—C19B—O2B	−179.39 (18)
C15A—C14A—C19A—O2A	−178.5 (2)	C17B—C18B—C19B—C14B	−1.5 (3)
C13A—C14A—C19A—O2A	−4.1 (3)	C15B—C14B—C19B—O2B	−179.88 (17)
C17A—C18A—C19A—C14A	0.6 (4)	C13B—C14B—C19B—O2B	8.8 (3)
C17A—C18A—C19A—O2A	178.6 (2)	C15B—C14B—C19B—C18B	2.3 (3)
C20A—O2A—C19A—C14A	−27.2 (3)	C13B—C14B—C19B—C18B	−169.04 (18)
C20A—O2A—C19A—C18A	154.9 (2)	C19B—O2B—C20B—C27B	166.38 (15)
C19A—O2A—C20A—C27A	−168.56 (18)	C19B—O2B—C20B—C21B	−61.74 (19)
C19A—O2A—C20A—C21A	59.2 (2)	O3B'—N2B—C21B—C13B	−22.4 (14)
O3A'—N2A—C21A—C20A	−99.6 (17)	O4B—N2B—C21B—C13B	−142.6 (5)
O4A—N2A—C21A—C20A	25.4 (15)	O3B—N2B—C21B—C13B	43.6 (4)
O4A'—N2A—C21A—C20A	57.6 (6)	O4B'—N2B—C21B—C13B	−173.4 (9)
O3A—N2A—C21A—C20A	−158.5 (6)	O3B'—N2B—C21B—C20B	97.7 (14)
O3A'—N2A—C21A—C13A	20.6 (17)	O4B—N2B—C21B—C20B	−22.4 (6)
O4A—N2A—C21A—C13A	145.6 (15)	O3B—N2B—C21B—C20B	163.7 (4)
O4A'—N2A—C21A—C13A	177.8 (6)	O4B'—N2B—C21B—C20B	−53.3 (9)
O3A—N2A—C21A—C13A	−38.3 (6)	O3B'—N2B—C21B—C22B	−136.4 (14)
O3A'—N2A—C21A—C22A	135.6 (17)	O4B—N2B—C21B—C22B	103.4 (6)
O4A—N2A—C21A—C22A	−99.4 (15)	O3B—N2B—C21B—C22B	−70.4 (4)
O4A'—N2A—C21A—C22A	−67.2 (6)	O4B'—N2B—C21B—C22B	72.5 (9)
O3A—N2A—C21A—C22A	76.7 (6)	C14B—C13B—C21B—N2B	96.99 (19)
O2A—C20A—C21A—N2A	60.0 (2)	C12B—C13B—C21B—N2B	−130.54 (16)
C27A—C20A—C21A—N2A	−67.0 (2)	C14B—C13B—C21B—C20B	−23.9 (2)
O2A—C20A—C21A—C13A	−59.9 (2)	C12B—C13B—C21B—C20B	108.61 (16)
C27A—C20A—C21A—C13A	172.98 (18)	C14B—C13B—C21B—C22B	−145.63 (15)
O2A—C20A—C21A—C22A	−176.09 (16)	C12B—C13B—C21B—C22B	−13.16 (18)
C27A—C20A—C21A—C22A	56.8 (3)	O2B—C20B—C21B—N2B	−59.52 (19)
C14A—C13A—C21A—N2A	−91.0 (2)	C27B—C20B—C21B—N2B	67.6 (2)
C12A—C13A—C21A—N2A	137.88 (18)	O2B—C20B—C21B—C13B	59.2 (2)
C14A—C13A—C21A—C20A	29.2 (2)	C27B—C20B—C21B—C13B	−173.75 (16)
C12A—C13A—C21A—C20A	−101.90 (18)	O2B—C20B—C21B—C22B	176.12 (14)
C14A—C13A—C21A—C22A	150.20 (17)	C27B—C20B—C21B—C22B	−56.8 (2)
C12A—C13A—C21A—C22A	19.04 (19)	C26B—N1B—C22B—C23B	−62.0 (2)
C12A—N1A—C22A—C23A	−163.08 (17)	C12B—N1B—C22B—C23B	166.59 (16)
C26A—N1A—C22A—C23A	63.2 (2)	C26B—N1B—C22B—C21B	168.41 (15)
C12A—N1A—C22A—C21A	−33.18 (19)	C12B—N1B—C22B—C21B	37.05 (18)
C26A—N1A—C22A—C21A	−166.94 (16)	N2B—C21B—C22B—N1B	102.47 (18)
N2A—C21A—C22A—N1A	−110.9 (2)	C13B—C21B—C22B—N1B	−13.01 (18)
C20A—C21A—C22A—N1A	125.88 (17)	C20B—C21B—C22B—N1B	−132.74 (16)
C13A—C21A—C22A—N1A	6.84 (19)	N2B—C21B—C22B—C23B	−21.0 (3)
N2A—C21A—C22A—C23A	12.5 (3)	C13B—C21B—C22B—C23B	−136.53 (18)
C20A—C21A—C22A—C23A	−110.7 (2)	C20B—C21B—C22B—C23B	103.8 (2)
C13A—C21A—C22A—C23A	130.2 (2)	N1B—C22B—C23B—C24B	56.4 (2)
N1A—C22A—C23A—C24A	−56.5 (3)	C21B—C22B—C23B—C24B	176.73 (18)
C21A—C22A—C23A—C24A	−177.1 (2)	C22B—C23B—C24B—C25B	−53.5 (3)
C22A—C23A—C24A—C25A	53.1 (3)	C23B—C24B—C25B—C26B	54.6 (3)
C23A—C24A—C25A—C26A	−53.5 (3)	C22B—N1B—C26B—C25B	60.7 (2)
C12A—N1A—C26A—C25A	170.03 (19)	C12B—N1B—C26B—C25B	−173.01 (17)
C22A—N1A—C26A—C25A	−61.1 (2)	C24B—C25B—C26B—N1B	−56.2 (3)
C24A—C25A—C26A—N1A	54.9 (3)	O2B—C20B—C27B—C28B	34.6 (3)
O2A—C20A—C27A—C28A	−35.0 (3)	C21B—C20B—C27B—C28B	−92.5 (2)
C21A—C20A—C27A—C28A	92.5 (3)	O2B—C20B—C27B—C32B	−144.46 (19)
O2A—C20A—C27A—C32A	143.4 (2)	C21B—C20B—C27B—C32B	88.5 (2)
C21A—C20A—C27A—C32A	−89.2 (3)	C32B—C27B—C28B—C29B	−3.8 (3)
C32A—C27A—C28A—C29A	3.1 (3)	C20B—C27B—C28B—C29B	177.15 (19)
C20A—C27A—C28A—C29A	−178.6 (2)	C27B—C28B—C29B—C30B	0.8 (3)
C27A—C28A—C29A—C30A	−0.9 (4)	C28B—C29B—C30B—C31B	1.8 (4)
C28A—C29A—C30A—C31A	−1.2 (4)	C28B—C29B—C30B—Cl2	177.0 (6)
C28A—C29A—C30A—Cl1A	−179.08 (18)	C28B—C29B—C30B—Cl2'	−170.1 (5)
C29A—C30A—C31A—C32A	1.0 (4)	C29B—C30B—C31B—C32B	−1.3 (4)
Cl1A—C30A—C31A—C32A	178.90 (18)	Cl2—C30B—C31B—C32B	−176.9 (6)
C30A—C31A—C32A—C27A	1.3 (4)	Cl2'—C30B—C31B—C32B	169.7 (6)
C28A—C27A—C32A—C31A	−3.3 (3)	C28B—C27B—C32B—C31B	4.2 (3)
C20A—C27A—C32A—C31A	178.3 (2)	C20B—C27B—C32B—C31B	−176.6 (2)
C4A—C5A—C33A—C34A	1.0 (4)	C30B—C31B—C32B—C27B	−1.8 (4)
C5A—C33A—C34A—C35A	−0.2 (4)	C4B—C5B—C33B—C34B	−0.4 (4)
C33A—C34A—C35A—C3A	−0.4 (4)	C5B—C33B—C34B—C35B	0.8 (5)
N3A—C3A—C35A—C34A	178.4 (2)	C33B—C34B—C35B—C3B	0.5 (4)
C4A—C3A—C35A—C34A	0.0 (3)	N3B—C3B—C35B—C34B	178.9 (2)
C4B—N4B—C1B—C2B	−1.0 (3)	C4B—C3B—C35B—C34B	−2.0 (4)

### Hydrogen-bond geometry (Å, º)

*Cg*1, *Cg*2 and *Cg*3 are the centroids of rings C14*B*–C19*B*, C3*B*–C5*B*/C33*B*–C35*B* and C3*A*–C5*A*/C33*A*–C35*A*, respectively.

**Table:**

*D*—H···*A*	*D*—H	H···*A*	*D*···*A*	*D*—H···*A*
C20*A*—H20*A*···N4*A*	0.98	2.28	3.170 (3)	151
C20*B*—H20*B*···N4*B*	0.98	2.45	3.298 (3)	144
C33*B*—H33*B*···O3*B*^i^	0.93	2.46	3.271 (5)	145
C8*A*—H8*A*···*Cg*1^ii^	0.93	2.69	3.536 (3)	151
C25*B*—H25*C*···*Cg*2^iii^	0.97	2.73	3.629 (3)	155
C31*B*—H31*B*···*Cg*3^iv^	0.93	2.99	3.742 (3)	139

Symmetry codes: (i) −*x*, *y*+1/2, −*z*+3/2; (ii) −*x*+1, *y*+1/2, −*z*+3/2; (iii) −*x*, −*y*, −*z*+2; (iv) *x*−1, *y*, *z*.
